# Recurrent spinal cord compression due to extramedullary hematopoiesis in thalassemia patient: Case report

**DOI:** 10.1097/MD.0000000000029334

**Published:** 2022-06-24

**Authors:** Shahem Abbarh, Abdulrahman F. Al-Mashdali, Mohamed Abdelrazek, Venkada Manickam Gurusamy, Mohamed A. Yassin

**Affiliations:** a Internal Medicine Department, Hamad Medical Corporation, Doha, Qatar; b Radiology Department, Hamad Medical Corporation, Doha, Qatar; c Radiation Oncology Department, Hamad Medical Corporation, Doha, Qatar; d Hematology and Oncology Department, Hamad Medical Corporation, Doha, Qatar.

**Keywords:** betal-thalassemia, extramedullary hematopoiesis, luspatercept, radiotherapy, spinal cord compression

## Abstract

**Introduction:**

Spinal cord compression secondary to extramedullary hematopoiesis (EMH) is a rare condition. Variable treatment options have been reported with different efficacy and recurrence rate. Due to its rarity, no clear optimal management guidelines have been established yet.

**Patient concerns and diagnosis:**

We report a recurrence of spinal cord compression secondary to EMH in a 19-year-old male, with a background of transfusion-dependent beta-thalassemia on luspatercept, who presented with weakness in both lower limbs.

**Interventions and outcomes:**

He was treated successfully both times with radiotherapy.

**Conclusion:**

Early diagnosis and management of EMH compressing the spinal cord are essential to prevent permanent neurological damage. Diagnosis should be suspected based on the clinical presentation and magnetic resonance imaging findings in a patient with a history of ineffective hemopoiesis. Treatment option remains controversial. Radiotherapy option seems effective, even in recurrent cases, and valid, particularly for those at high risk of surgery or who do not prefer it.

## 1 Introduction

Thalassemia is a hereditary disease characterized by defective hemoglobin synthesis leading to ineffective erythropoiesis. Depending on its severity, thalassemia may present with different clinical features and can be classified into transfusion-dependent and transfusion-independent.^[1]^ With increasing age, even patients with mild forms may develop complications from iron deposition in tissues,^[2]^ with many subsequent health problems such as growth retardation,^[3]^ fertility issues,^[4]^ and hypoadrenalism,^[5]^ which require chelation therapy.^[6]^ Less frequently, patients may also develop complications secondary to massive erythroid expansion (known as extramedullary hematopoiesis “EMH”).^[7]^

EMH is defined as a physiological compensatory production of red blood cells in tissues other than bone marrow secondary to chronic anemia. It is typically seen in organs vigorously involved in fetal hematopoiesis, including liver, spleen, and lymph nodes.^[8,9]^ Rarely, it can also be seen in other sites including epidural space, adrenal, pleural, and thyroid.^[10,11]^ EMH in epidural space is a rare and particularly sensitive location as it can cause spinal compression and may present as a paravertebral pseudotumor presenting as back pain and neurological disorder of the lower limbs. Treatment options vary and include blood transfusions, radiotherapy, and decompressive surgery, with different responses and recurrence rates. Due to its rarity, with only a few case reports and case series have been published, no guidelines for optimal management of such cases have been established to date. A new drug called luspatercept was found to decrease transfusion requirements by improving red blood cells maturation, and therefore, it may downregulate the EMH process. It was approved for treating adults with transfusion-dependent beta-thalassemia in November 2019.^[12]^ However, the effect on the process of EMH has not been adequately assessed in the literature. Herein, we present a case of recurrent spinal cord compression due to EMH in a young patient with β-thalassemia major on luspatercept after being treated the first time successfully with radiotherapy.

## 2 Case presentation

A 19-year-old man with a background of β-thalassemia major presented with a two-week history of bilateral lower limbs weakness. The weakness was progressive to a limit he had to use a cane for assistance during the last 3 days. This was also associated with difficulty in walking and urinary urge incontinence. He denied other symptoms or trauma with a normal bowel motion. He was on hydroxyurea, regular blood transfusion every 3 weeks along with iron chelation therapy, and luspatercept. The latter was started 6 weeks prior to presentation at a 1 mg/kg dose, with only 2 doses received. Notably, the patient had a similar presentation around 15 months ago when he was diagnosed with spinal cord compression secondary to EMH in the thoracic spine (T2-T9 level). He received ten fractions of radiotherapy with a dose of 2000 cGy over 14 days with significant improvement. After that, he was back to his baseline and continued to receive hydroxyurea and regular blood transfusions to prevent recurrence. Despite clinical improvement, there was no remarkable improvement on the follow-up Magnetic resonance imaging (MRI) spine after eight months of completing the radiotherapy sessions.

Physical examination of the lower limbs showed a power of 3/5 bilaterally in the proximal and distal groups as well as spasticity, hyperreflexia with clonus, and positive bilateral Babinski reflexes. There was a sensory level at the T8/9 level. Cranial nerves and upper limb examination both were completely normal. Laboratory investigations revealed a hemoglobin (Hb) level of 10.7 gm/dL, with normal white blood cell count and platelets. Ferritin was 5260 ug/L. Total bilirubin was 76 umol/L, with indirect bilirubin of 69 umol/L. C-reactive protein and erythrocyte sedimentation rate were negative.

**Figure 1:**
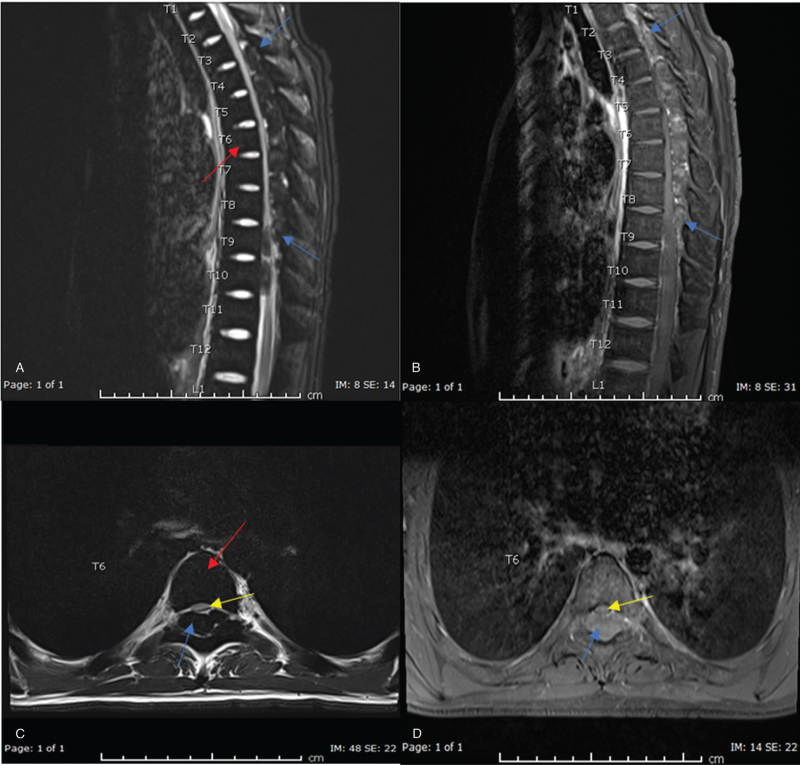
Extra medullary hematopoiesis extending from T2 down to T9 level with extensive intraspinal posterior epidural component (Blue arrows in A, B, C &D) causing moderate to marked cord compression (yellow arrows in C& D). Note the dark signal of the bone marrow of thoracic spine in T2 image (red arrows in A & C) due to chronic hemolytic anemia. (A) Sagittal T2 weighted image of thoracic spine. (B) Sagittal post contrast fat saturated image of thoracic spine. (C) Axial T2 weighted image of thoracic spine at T6 level. (D) Axial post contrast fat saturated image of thoracic spine at T6 level.

MRI spine revealed EMH process extending from T2 down to T9 level with extensive intraspinal posterior epidural component causing moderate to severe cord compression as shown in Figure 1. These changes were similar to the changes seen in a previous MRI done during the patient's first presentation. In the context of the patient's presentation, his background of thalassemia, and the MRI findings, the diagnosis of recurrent spinal cord compression due to EMH secondary to thalassemia was established.

The patient was started on dexamethasone (4 mg oral TID) and baclofen. In addition to the regular blood transfusion (every 3 weeks), he received 2 additional blood transfusions during his hospital stay to keep Hb >10. A multi-disciplinary team meeting was scheduled; however, the patient showed rapid deterioration in his clinical status (power in hip flexors decreased to 1/5). He was offered surgical intervention (decompression with instrumentation and possible debulking) as it recurred after managing it with radiotherapy the first time. However, the patient declined the surgical option and preferred to receive radiotherapy again. Thus, ten fractions of radiotherapy (2000 cGy) were delivered over 12 days. Moreover, he was on daily sessions of physiotherapy and occupational therapy. There was a significant improvement of the weakness after completing the radiotherapy sessions, with lower limbs power improved to +4/5. No complications of radiotherapy were noticed. The patient was discharged on his usual regular blood transfusion along with physiotherapy sessions. Luspatercept was continued as well. On follow up, 1 month after completing the radiotherapy, he was back to his baseline with normal gait. The patient provided an informed consent, and the study protocol was approved by the institutional review board of our hospital.

## 3 Discussion

EMH is characterized by the formation of hematopoietic elements outside the bone marrow. It occurs as a compensatory sequela of persistently ineffective hemopoiesis or impaired bone marrow function. Therefore, it can be seen in patients with different causes of chronic anemia, including thalassemia, congenital hemolytic anemia, hemoglobinopathies, and myelofibrosis. Although patients with intermedia thalassemia tend to have a milder form of the disease, both thalassemia major and intermedia can provoke EMH. The transfusion-independence in patients with thalassemia intermedia may burden the compensatory mechanisms leading eventually to extensive EMH.

Despite being a noncancerous physiologic reaction, EMH may cause a mass effect, depending on the location and mass volume. Spinal cord compression due to EMH in the paraspinal region is a true rarity. Since its description by Gatto et al in 1954,^[13]^ only a handful of case reports and series have been reported in the literature. It usually has a predilection for the lower thoracic spine where the limited mobility and narrow spinal canal predispose itself to spinal cord compression.^[14,15]^ The clinical presentation may mimic paravertebral tumors, including back pain, paraparesis, paresthesia in the lower limbs, gait instability, and sphincter disturbances.^[16]^ Complete paraplegia of the lower limbs has also been reported.^[17]^

Rapid diagnosis and treatment of this condition are essential to optimize the chances of recovery and prevent irreversible neurological damage. Unfortunately, the rarity of the disease and the nonspecific findings usually hinder the initial consideration of the diagnosis. Therefore, a history of a disease resulting in chronically ineffective hemopoiesis should prompt the physician to have a high index of suspicion. The diagnostic procedure of choice is MRI.^[18]^ Imaging of epidural extramedullary hematopoiesis reveals lesions that vary in intensity and contrast enhancement depending on age and the hematopoietic activity of the lesion.^[19]^ Biopsy is not always required for diagnosis, and most authors do not favor a tissue biopsy in such conditions.^[15,20]^ For our patient, the diagnosis was made based on the thalassemia background, history of weakness in the lower limbs, combined with the MRI findings.

Different treatment options of spinal cord compression secondary to EMH have been reported in the literature, including multiple transfusions to downregulate the EMH process, radiation therapy, surgical decompression, or any combination of the above treatments. Unfortunately, because of its infrequency, no strong evidence-based guidelines for the treatment of such condition have been established. In general, therapy choice depends on the severity of symptoms, mass size, patient preference, and previous treatment.

The role of transfusion is typically an adjunctive therapy while waiting for more definitive treatment.^[16]^ Nevertheless, several cases in the literature were managed successfully with solely blood transfusion. Parsa and Oriezy argued that transfusion therapy could be used as the sole treatment modality in patients with thalassemia.^[21]^ Emamhadi and Alizadeh reported that their patient had rapid neurological recovery within days and complete resolution of the EMH mass on MRI within 1 week after being treated with hypertransfusion alone. However, it was not mentioned how many units were transfused and the subsequent level of Hb after hypertransfusion.^[22]^ Similarly, Aliberti et al described 2 cases of paraparesis due to EMH mass compressing the spinal cord that was successfully managed by blood hypertransfusion. The first patient was transfused 24 units of packed red blood cells (1 unit every 10 days) with Hb increasing from 11.2 g/dL to 16 g/dL. The second patient received 16 units to raise Hb level from 10 g/dL to 14.5 g/dL. Furthermore, both patients showed no recurrence during a four-year follow-up period.^[23]^ In general, there is no consensus in the current literature regarding the ideal Hb level for treatment with blood transfusion. The drawbacks of blood transfusions include requirements for intravenous access, time spent receiving the transfusion, potential for transfusion reactions or infectious disease transfer, and iron overload. For adults with transfusion-dependent beta-thalassemia, luspatercept is a new means of reducing these burdens.

Luspatercept (previously called ACE-536) is a subcutaneous agent, given every 3 weeks, which was approved to treat transfusion-dependent beta-thalassemia in November 2019.^[12]^ Its mechanism of action is partially understood and involves sequestering of activin A, which contributes to TGF-beta signaling, eventually leading to improvement of red blood cell maturation. The primary evidence for the efficacy of luspatercept comes from the BELIEVE trial, published in early 2020.^[24]^ In that trial, luspatercept significantly reduced transfusion requirements compared with placebo with a reduction in transfusions by one-third and at least 2 units during a 12-week period (71 vs 30 percent). As luspatercept decreases the transfusion requirements, it may also decrease the need for the compensatory EMH process. To date, the role of luspatercept in suppressing the EMH process has not been studied thoroughly. In our literature review, there are no reported cases of spinal cord compression due to EMH in a thalassemia patient while on luspatercept. Our patient was started on luspatercept 6 weeks before the presentation, with only 2 doses received. Therefore, it was early to assess its effect on our patient's blood transfusion requirement. It is unknown if the continuation of this drug for a more extended period will help in preventing a recurrence of spinal cord compression by increasing the Hb and suppressing EMH.

In order to halt the production of overgrown marrow tissue, radiation therapy has been used in many cases of EMH mass either as monotherapy^[15,17,25,26]^ or combined with surgery^[14,27,28]^ with good results. Some authors even suggested that radiation therapy should be the primary treatment modality with surgical intervention reserved for recurrent cases postradiation.^[29]^ Improvement is clinically evident after an average of 3 fractions of radiotherapy, and near-complete recovery is generally observed by the end of treatment.^[29]^ The optimal dose of radiation in this setting has not been established in proper dose-response studies; however, hematopoietic tissue is known to be very radiosensitive. The radiation dosage used in previous cases has ranged from as low as 750 cGy to 3500 cGy.^[16,30]^ Our patient received ten radiotherapy sessions of 2000 cGy with complete resolution of symptoms during the first episode, which was well below the spinal cord radiation tolerance dose, estimated at 5000 cGy.^[31]^ Hence, he was offered reirradiation during recurrence. Unfortunately, radiotherapy also carries risks, including possible deterioration of neurologic function due to tissue edema, bone marrow suppression, and long-term side effect of radiotherapy.^[14]^ Additionally, although many cases reported complete resolution of symptoms within days of starting radiotherapy, several cases, including our case, showed recurrence. The reported recurrence rates in the literature range between 19% and 37%.^[17,30]^

Surgical intervention in patients with EMH mass compressing the spinal cord involves laminectomy with excision of the EMH mass. It can provide immediate relief to the compressed cord, thus improving neurological recovery. At the same time, an accurate histologic diagnosis can be made.^[19]^ Salehi SA et al argued that surgery is preferred as the first-line treatment, particularly in young individuals, as it offers both immediate decompression of the spinal cord and avoids long-term effects of radiotherapy.^[16]^ Disadvantages of surgical option include risk of bleeding associated with the high vascularity of the mass, risks of operating on anemic individuals who are predisposed to shock, incomplete excision in cases of diffuse involvement,^[32]^ and instability of the spine after multi-levels laminectomy.^[33]^ Unfortunately, recurrence was also seen after decompression surgery.^[14]^ After all, the lack of treatment guidelines presents a challenge in treating such conditions with no modality of treatment that has been proven superior to another.

## 4 Conclusion

Extensive EMH in the epidural space causing spinal cord compression and consequent neurological problems is a rare condition. Early diagnosis and treatment are essential to prevent progression and permanent neurological damage. Diagnosis should be suspected based on the clinical presentation and MRI findings in a patient with a background of ineffective hemopoiesis. To date, treatment remains controversial, with no guidelines have yet been proposed. However, radiotherapy seems effective, even in recurrent cases, and a valid option, particularly for those at high risk of surgery or who do not prefer it.

## Author contributions

Shahem Abbarh: Writing – original draft; Data curation

Abdulrahman F. Al-Mashdali: Writing – review and editing

Mohamed Abdelrazek: Writing – review and editing; Visualization

Venkada Manickam Gurusamy: Writing – review and editing

Mohamed A. Yassin: Writing – review and editing; Supervision

All authors approved the final version of the manuscript.

**Conceptualization:** Mohamed Yassin, Shahem Abbarh.

**Data curation:** Abdulrahman Al-Mashdali, Mohamed Yassin, Shahem Abbarh.

**Supervision:** Mohamed Yassin.

**Visualization:** Mohamed Abdelrazek.

**Writing - original draft:** Shahem Abbarh.

**Writing - review & editing:** Abdulrahman Al-Mashdali, Mohamed Abdelrazek, Mohamed Yassin, Venkada Gurusamy.
